# Association between soft drink consumption and cardiovascular disease risk among Brazilian adults: a cross-sectional study

**DOI:** 10.1590/1516-3180.2023.0433.R1.29112024

**Published:** 2025-08-25

**Authors:** Karla Cordeiro Gonçalves, Luís Antônio Batista Tonaco, Guilherme Augusto Veloso, Alexandra Dias Moreira, Mariana Santos Felisbino-Mendes, Deborah Carvalho Malta, Gustavo Velasquez-Melendez

**Affiliations:** I Nurse, Doctoral student, Postgraduate Program in Nursing, School of Nursing, Universidade Federal de Minas Gerais (UFMG), Belo Horizonte (MG), Brazil.; II Nurse, Post-Doctoral Student, Postgraduate Program in Nursing, Universidade Federal de Minas Gerais (UFMG), Belo Horizonte (MG), Brazil.; III Statistical, Assistant Professor, Statistics Department, Universidade Federal Fluminense (UFF), Niteroi (RJ), Brazil.; IV Nurse, Assistant Professor, Department of Maternal-Child Nursing and Public Health, School of Nursing, Universidade Federal de Minas Gerais (UFMG), Belo Horizonte (MG), Brazil.; V Nurse, Assistant Professor, Department of Maternal-Child Nursing and Public Health, School of Nursing, Universidade Federal de Minas Gerais (UFMG), Belo Horizonte (MG), Brazil.; VI Physician, Associate Professor and researcher, School of Nursing, Universidade Federal de Minas Gerais (UFMG), Belo Horizonte (MG), Brazil.; VII Biologist, Professor, Department of Maternal-Child Nursing and Public Health, School of Nursing, Universidade Federal de Minas Gerais (UFMG), Belo Horizonte (MG), Brazil.

**Keywords:** Cardiovascular diseases., Risk assessment., Sugar-sweetened beverages., Artificially sweetened beverages., Health surveys., Brazil., Sugar-sweetened soft drinks., Ultra-processed food., Food consumption., Cardiovascular risk estimation.

## Abstract

**BACKGROUND:**

Inadequate diet is considered a major risk factor for chronic noncommunicable diseases and mortality. Among the ultra-processed foods, sweetened soft drinks are significant contributors to high-calorie diets and are associated with adverse health outcomes.

**OBJECTIVE:**

To estimate the association between soft drink consumption and the risk of cardiovascular events.

**DESIGN AND SETTING:**

A cross-sectional study was conducted using data of adults aged ≥ 18 years from the 2013 National Health Survey.

**METHODS:**

The explanatory variable was the daily consumption of sugar-sweetened soft drinks. Cardiovascular risk (CVR) was calculated using the Framingham score. Multinomial logistic regression was used for the analyses. Two models were used: one adjusted for age and body mass index and the other for age and waist circumference. Both models were applied to the general population and stratified by race and educational attainment.

**RESULTS:**

The study sample consisted of 8,391 participants. Individuals with sugary soda consumption ≥ 0.4 cups/day were associated with a higher CVR, which escalated with increasing consumption of soft drinks.

**CONCLUSIONS:**

CVR was observed across all consumption categories and difference in risk was based on the intake quantity.

## INTRODUCTION

 The concept of diet quality reflects an evaluation of various food types, nutrients, and dietary constituents in relation to established dietary recommendations and/or health outcomes, which can be verified through diet quality indices.^1^ Inadequate diet is considered a major risk factor for chronic noncommunicable diseases and mortality.^2^ Among foods that undergo a high degree of industrial processing, ultra-processed foods are considered the most harmful to health and their consumption is associated with an increased incidence of diseases.^3^

 Sweetened soft drinks, classified as ultra-processed foods, account for a large proportion of high-calorie diets, which are associated with excessive weight gain.^4^

 These beverages are consumed by a considerable proportion of the Brazilian population.^5^ Consumption patterns differ among sociodemographic, regional, and economic variables of the population.^5,6^

 Evidence that increased soft drink consumption is associated with adverse health outcomes is not recent.^7^ These beverages are typically sweetened with corn syrup or sucrose; a component of these sugars is the monosaccharide fructose which, when consumed frequently, results in increased visceral and intramuscular fat deposition,^8^ elevated levels of uric acid,^9^ insulin resistance, and dyslipidemia.^10^

 Ultra-processed and sugar-sweetened beverages such as soft drinks increase the risk of obesity, type 2 diabetes, hypertension, and all-cause mortality.^11^ They are also associated with a higher incidence of metabolic syndrome and its components.^12^ Results from a cohort of Mexicans showed that participants in the highest quintile of refined food consumption, which included soft drink consumption, had a 10% higher cardiovascular risk (CVR) over 10 years than those in the lowest quintile of consumption.^13^ A meta-analysis of prospective studies revealed that soft drink intake was associated with a dose-dependent increase in the risk of acute myocardial infarction and stroke.^14^

 Previous analyses have been reported an association between soft drink consumption and CVR factors, such as hypertension and obesity, in the Brazilian population.^12,15 ,16^ However, to our knowledge, no previous studies have analyzed the association between the consumption of these beverages and the risk of cardiovascular events in adults using a representative sample of the Brazilian population. This knowledge is essential for the strengthening prevention and health promotion measures, encouraging healthy eating patterns, and guiding public policies aimed at controlling soft drink consumption, with a focus on reducing future cardiovascular events in the Brazilian population. 

## OBJECTIVE

 This study aimed to estimate the association between soft drink consumption and the risk of cardiovascular events. We conducted a national survey of a representative sample of the Brazilian population. Thus, we tested the hypothesis of an association between an increased consumption of sugar-sweetened beverages and an elevated cardiovascular disease risk. 

## METHODS

### Design and study population

 This cross-sectional study was based on data from the 2013 National Health Survey (NHS), with adults aged ≥ 18 years. Data from laboratory tests collected in 2014 and 2015 were used and the two databases were correlated. The survey refers to the first laboratory edition of the NHS conducted in 2014 and 2015, which is the only Brazilian survey to collect laboratory tests from a representative sample of the national population, resulting in greater accuracy of the estimates.^17^ The methodology of the NHS sampling process and the sub-sample for laboratory data collection is detailed in previous studies.^ 17-19^

 The exclusion criteria for this study were the same as those adopted for the construction of the CVR estimates proposed by D’Agostino et al. These criteria excluded individuals aged < 30 years and > 74 years as well as individuals who reported being diagnosed with heart disease or stroke.^20^

 After applying the exclusion criteria, we obtained a sample of 8,391 individuals. 

### Laboratory data collection

 The collection of biological material was performed at the homes of the participants after they received an explanation of the procedure and signed the consent form for collection.^17^ After blood collection, the samples were centrifuged and the resulting serum and plasma were stored in refrigerators at 4^°^C; the entire process was performed using calibrated equipment.^21^

 The laboratory tests performed on the blood samples in this study included glycated hemoglobin, total cholesterol, and low- and high-density lipoprotein (LDL and HDL, respectively) levels. 

### Study variables

 The dependent variable, CVR at 10 years, was constructed according to the Framingham score criteria.^20^ The variables HDL, total cholesterol, treated-, and untreated- systolic blood pressure were considered, in numerical categories. Diabetes and smoking status were self-reported (yes/no). All the variables were adjusted for sex. Further details and cutoff points for each variable were specified as described by D’Agostino et al.^20^

 The 10-year CVR categories were low CVR < 5%, medium CVR 5–20%, and high CVR ≥ 20%, based on the guidelines of the Brazilian Society of Cardiology.^22^

 The explanatory variable in this study was the daily consumption of a standard portion (1 cup) of sugary drinks based on the following question: "*How many days a week do you usually drink soda (or artificial juice)?*" The responses were categorized as follows: < 0.1 cup/day, 0.1–0.39 cups/day, 0.4–0.99 cups/day, and ≥ 1.0 cups/day.^9^

 The sociodemographic variables used were sex (male, female), age (30–74 years), race/skin color grouped into either White or Other (black, yellow, brown, and indigenous), and educational level categorized as low (individuals without formal education to those who did not complete middle school) and high (individuals who completed middle school, did not complete high school, and completed high school). 

 Physical activity levels were recorded based on the following questions: "*How many days a week do you usually practice physical exercises or sports?*"; "*In general, on the day you usually practice exercise or sports, how many hours does this activity last?*"; and "*In general, on the day you usually practice exercise or sports, how many minutes does this activity last?*" Physical activity was defined as at least 150 min. of moderate activity per week or 75 min. of vigorous activity per week, during leisure time.^23^

 The variable smoking (non-smoker, ex-smoker, and smoker) was created from the following questions: "*Do you currently smoke any tobacco products?*" and " *In the past, have you smoked any tobacco products daily?* "^18,24^

 The variable alcohol consumption (no, light/moderate, and heavy drinking) was constructed based on the following questions: "*How often do you usually consume alcoholic beverages?*" and "*In the past 30 days, have you consumed 5 or more drinks on a single occasion?* "^23^ Habitual drinking (drinking in the past 30 days, regardless of dose) and heavy drinking (drinking five or more drinks for men and four or more drinks for women on a single occasion in the past 30 days) were considered.^18^

 Regarding anthropometric variables, body mass index (BMI) was categorized as underweight (< 18.5 kg/m^2^), eutrophic (18.525 kg/m^2^), overweight (25–30 kg/m2), and obese (≥ 30 kg/m2). Altered waist circumference (WC) constituted "no" (< 88 cm in women and < 102 cm in men) and "yes" (≥ 88 cm in women and ≥ 102 cm in men).^24^

 Hypertension was defined with a systolic blood pressure ≥ 140 mmHg or a diastolic blood pressure ≥ 90 mmHg or use of antihypertensive medications,^25,26^ obtained in response to the question, "In the past two weeks, have you taken medication for hypertension (high blood pressure)?"^23^ In all, three measurements were obtained at 2-min intervals; subsequently, the mean of the three readings was recorded as the definitive value for data analysis.^21^

 Finally, laboratory variables included altered HDL cholesterol (≤ 40 mg/dL in men and ≤ 50 mg/dL in women), altered total cholesterol (≥ 200 mg/dL), altered LDL cholesterol (≥ 130 mg/dL),^24^ and the diagnosis of diabetes when individuals presented HbA1c levels ≥ 6.5%.^27,28^

### Data analysis

 Initially, descriptive analysis was performed using the relative frequency of the data and the chi-square test for comparison of proportions according to sociodemographic variables, lifestyle and anthropometric and laboratory measurements. 

 Next, multivariate regression was performed using multinomial logistic regression to verify the association between the explanatory variables and the 10-year CVR variables in the three categories (low, medium, and high CVR). The association between sugar-sweetened beverage consumption and CVR was estimated using odds ratios (OR) and 95% confidence interval (95%CI). The reference category of the exposure variable used for comparison between analyses was soft drink consumption < 0.1 cup/day. 

 Two models were used: one adjusted for age and BMI, and the other for age and WC. The adjustment variables were selected based on the literature,^11,29 -32^ specifically BMI and WC, because the other variables suggested in the literature were already part of the proposed CVR score. The models were run for the general population and stratified by skin color/race (White and Other) and education (low/high) owing to differences in racial admixture and socioeconomic factors presented in the study population that differed from the population originally used for the Framingham score. 

 Data analyses were performed using the Stata 14.0 software (Stata Corp., College Station, Texas, United States) in the *survey* module, which included complex sample structure data for population estimates. 

### Ethical aspects

 The NHS was approved by the National Research Ethics Committee of the Brazilian Ministry of Health in July 2013. 

 Participation in the research was voluntary and information confidentiality was guaranteed. The research participants signed an informed consent form and authorized the collection of laboratory test results.^17,18,21^

## RESULTS

**Table 1** presents the distribution of clinical and sociodemographic profile variables according to soft drink consumption categories. The category with the highest consumption consisted predominantly of men (52.99%), individuals aged 30–39 years (40.11%), those with low educational levels (51.80%), and consumers of nonalcoholic beverage (70.83%). Additionally, in the high consumption category, there was a higher prevalence of nonsmokers (64.21%), individuals with normal weight (43.74%), normal WC (65.23%), normal lipid profile (total cholesterol, 71.43%; LDL, 84.16%), nondiabetics (94.00%), non-hypertensive individuals, and those with low CVR (65.39%). 

**Table 1:** Prevalences of the population by clinical and sociodemographic characteristics of the Brazilian adults in relation to consumption of sweetened soft drinks. National Health Survey, 2013.

**Variables**		**Soft drink consumption (cups/day)**				**P**
		**< 0.1**	**0.1–0.39**	**0.4–0.99**	**≥1.0**	
		**% (95% CI)**	**% (95%CI)**	**% (95%CI)**	**% (95%CI)**	
**Sex**						
	Male	38.44 (36.03–40.91)	43.52 (40.77–46.31)	58.56 (55.45–61.59)	52.99 (49.88–56.09)	<0.001
	Female	61.56 (59.09–63.97)	56.48 (53.69–59.23)	41.44 (38.41–44.55)	47.01 (43.91–50.12)	
**Age (years)**						
	30–39	19.11 (17.04–21.37)	29.10 (26.03–32.06)	35.43 (31.94–39.07)	40.11 (36.45–43.89)	<0.001
	40-49	26.27 (23–87–28.82)	26.86 (24.23–29.65)	28.32 (25.15–31.73)	29.12 (25.86–32.60)	
	50-59	26.18 (23–87–28.63)	23.78 (21.31–26.44)	23.27 (20.14–26.72)	19.81 (17.00–22.96)	
	60–74	28.44 (26.16–30.83)	20.26 (17.98–22.75)	12.98 (10.82–15.50)	10.96 (9.03–13.23)	
**Color**						
	White	50.42 (47.96–52.88)	47.19 (44.43–49.98)	45.72 (42.44–49.03)	48.55 (45.41–51.70)	0.2709
	Black	8.40 (07.15–09.85)	9.50 (8.01–11.23)	10.57 (08.69–12.80)	9.26 (7.52–11.35)	
	Yellow	0.98 (0.510–1.88)	0.38 (0.21–0.69)	0.66 (0.39–01.12)	0.49 (0.23–1.01)	
	Brown	39.89 (37.59–42.24)	42.58 (39.94–45.27)	42.78 (39.68–45.93)	41.38 (38.41–44.41)	
	Indigenous	0.30 (0.17–0.54)	0.34 (0.15–0.79)	0.27 (0.13–0.56)	0.33 (0.18–0.61)	
**Level of education**						
	Low	60.34 (57.82–62.82)	54.27 (51.47–57.04)	48.93 (45.70–52.17)	51.80 (48.66–54.93)	<0.001
	High	39.66 (37.18–42.18)	45.73 (42.96–48.53)	51.07 (47.83–54.30)	48.20 (45.07–51.34)	
**Physical activity**						
	Yes	21.14 (19.12–23.32)	22.03 (19.80–24.43)	22.94 (20.15–26.00)	21.83 (19.26–24.65)	0.7973
	No	78.86 (76.68–80.88)	77.97 (75.57–80.20)	77.06 (74.00–79.85)	78.17 (75.35–80.74)	
**Consumption of alcoholic beverages**						
	Non-drinker	80.40 (78.31–82.34)	79.36 (76.90–81.61)	68.12 (64.88–71.20)	70.83 (67.87–73.63)	<0.001
	Light/Moderate	14.49 (12.76–16.41)	16.91 (14.82–19.23)	23.93 (21.13–26.97)	19.24 (16.89–21.83)	
	Abusive	5.10 (4.15–6.26)	3.74 (2.80-4.98)	7.95 (6.25–10.06)	9.93 (8.12–12.08)	
**Smoking**						
	Non-smoker	65.15 (62.81–67.42)	70.12 (67.54–72.57)	70.13 (67.09–73.01)	64.21 (61.15–67.16)	0.0005
	Ex-smoker	19.58 (17.77–21.51)	17.93 (15.94–20.10)	15.45 (13.40–17.74)	17.93 (15.77–20.31)	
	Smoker	15.27 (13.60–17.11)	11.96 (10.25–13.90)	14.42 (12.16–17.02)	17.86 (15.48–20.53)	
**BMI**						
	Low weight	2.60 (1.94–3.49)	2.31 (1.59–3.32)	1.74 (1.08–2.80)	3.40 (2.27–5.06)	0.0041
	Eutrophic	36.65 (34.29–39.08)	38.44 (35.75–41.19)	42.21 (38.99–45.49)	43.74 (40.65–46.89)	
	Overweight	36.60 (34.28–38.99)	37.22 (34.63–39.88)	35.42 (32.42–38.55)	32.63 (29.83–35.57)	
	Obesity	24.14 (22.09–26.32)	22.04 (19.85–24.39)	20.63 (18.17–23.33)	20.22 (17.82–22.86)	
**Altered waist circumference**						
	Yes	46.45 (44.01–48.90)	41.28 (38.62–43.99)	32.77 (29.91–35.76)	34.77 (31.90–37.75)	<0.001
	No	53.55 (51.10–55.99)	58.72 (56.01–61.38)	67.23 (64.24–70.09)	65.23 (62.25–68.10)	
**Altered HDL cholesterol**						
	Yes	54.10 (51.63–56.55)	51.55 (48.78–54.31)	50.28 (47.02–53.53)	54.35 (51.20–57.47)	0.1600
	No	45.90 (43.45–48.37)	48.45 (45.69–51.22)	49.72 (46.47–52.98)	45.65 (42.53–48.80)	
**Altered total cholesterol**						
	Yes	36.95 (34.62–39.33)	33.50 (31.02–36.07)	30.94 (28.11–33.92)	28.57 (25.91–31.38)	<0.001
	No	63.05 (60.67–65.38)	66.50 (63.93–68.98)	69.06 (66.08–71.89)	71.43 (68.62–74.09)	
**Altered LDL cholesterol**						
	Yes	21.50 (19.57–23.57)	18.47 (16.65–20.56)	17.39 (15.20–19.83)	15.84 (13.80–18.12)	0.0013
	No	78.50 (76.43–80.43)	81.53 (79.44–83.45)	82.61 (80.17–84.80)	84.16 (81.88–86.20)	
**Diabetes (HbA1c > 6.5 or medication)**						
	Yes	12.62 (11.13–14.27)	8.41 (7.06–9.98)	6.44 (5.11–8.09)	6.00 (4.75–7.56)	<0.001
	No	87.38 (85.73–88.87)	91.59 (90.02–92.94)	93.56 (91.91–94.89)	94.00 (92.44–95.25)	
**Hypertension**						
	Yes	28.35 (26.16–30.64)	24.80 (22.49–27.25)	19.96 (17.61–22.55)	21.80 (19.28–24.54)	<0.001
	No	71.65 (69.36–73.84)	91.59 (90.02–92.94)	93.56 (91.91–94.89)	94.00 (92.44–95.25)	
**Cardiovascular risk**						
	Low	39.68 (37.25–42.17)	52.47 (49.72–55.20)	61.60 (58.47–64.65)	65.39 (62.42–68.24)	<0.001
	Medium	37.09 (34.76–39.47)	31.53 (29.08–34.09)	25.64 (22.99–28.49)	24.51 (21.94–27.27)	
	High	23.23 (21.30–25.28)	15.99 (14.17–18.00)	12.75 (10.87–14.90)	10.10 (8.55–11.90)	

^*^Pearson’s chi-square test; CI = confidence interval; BMI = body mass index; HDL = high-density lipoprotein; LDL = low-density lipoprotein.

**Figure 1:**
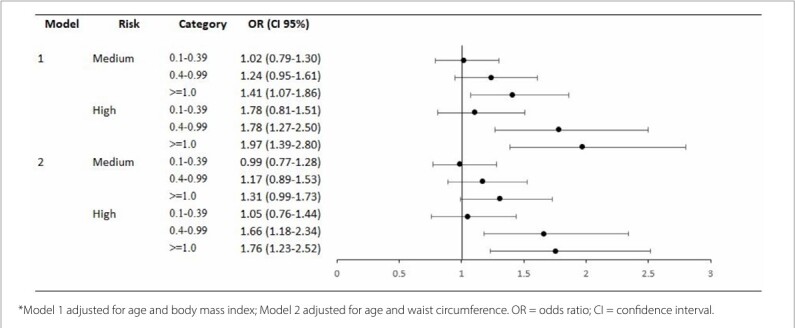
Odds ratio and 95% confidence interval for medium/high cardiovascular risk according to the consumption of daily servings of sweetened soft drinks in the Brazilian population. National Health Survey, 2013.

**Figure 1** shows the OR estimates for medium/high CVR. Soft drink consumption between 0.4–0.99 servings/day was associated with higher odds of high cardiovascular risk (CVR) scores in both the model adjusted for BMI (OR = 1.78; 95%CI = 1.27–2.50) and the model adjusted for WC (OR = 1.66; 95%CI = 1.18–2.34). Consumption of ≥ 1.0 serving/day was associated with higher odds of both medium (OR = 1.41; 95%CI = 1.07–1.86) and high CVR scores (OR = 1.97; 95%CI = 1.39–2.80) in the BMI-adjusted model. In the WC-adjusted model, this level of consumption was associated only with higher odds of high CVR scores (OR = 1.76; 95%CI = 1.23–2.52). 

**Table 2** presents the models stratified by skin color, race, and education. Overall, the significant association between soft drink consumption and CVR scores remained consistent in the ’Other’ race/skin color category, as well as in both high- and low-education strata. These models were adjusted for age BMI and WC. 

**Table 2:** Odds ratio and 95% confidence interval (95% CI) for medium/high 10-year risk score according to consumption of sweetened soft drinks in the adult Brazilian population stratified by race and education. National Health Survey, 2013.

	**Medium risk**				**High risk**			
	OR (95%CI)*	P*	OR (95%CI)**	P**	OR (95%CI)*	P*	OR (95%CI)**	P**
**Soft drink consumption (cups/day)**								
**White**								
**< 0.1**	Ref.		Ref.		Ref.		Ref.	
**0.1–0.39**	1.04 (0.71–1.51)	0.843	0.99 (0.68-1.46)	0.982	0.99 (0.61–1.58)	0.955	0.89 (0.55–1.45)	0.651
**0.4–0.99**	1.16 (0.77–1.75)	0.467	1.07 (0.70-1.62)	0.760	1.67 (**1.01–2.74**)	0.044	1.48 (0.88–2.47)	0.136
**≥ 1.0**	1.25 (0.82–1.91)	0.296	1.13 (0.74-1.72)	0.573	1.64 (0.97–2.78)	0.065	1.42 (0.82–2.45)	0.205
**Other**								
**< 0.1**	Ref.		Ref.		Ref.		Ref.	
**0.1–0.39**	0.97 (0.71–1.33)	0.861	0.97 (0.71-1.33)	0.857	1.21 (0.80–1.81)	0.365	1.19 (0.78–1.78)	0.016
**0.4–0.99**	1.26 (0.90–1.76)	0.179	1.22 (0.86–1.71)	0.259	1.83 (1.16–2.89)	0.009	**1.76 (1.11–2.79)**	0.016
**≥1.0**	1.60 (1.13–2.26)	0.018	1.52 (**1.07–2.14**)	0.018	2.45 (**1.55–3.87**)	<0.0001	**2.24 (1.41–3.57)**	0.001
**Low educational level**								
**< 0.1**	Ref.		Ref.		Ref.		Ref.	
**0.1–0.39**	0.89 (0.65–1.22)	0.468	0.89 (0.65–1.22)	0.466	0.94 (0.65–1.37)	0.766	0.92 (0.63–1.33)	0.646
**0.4–0.99**	1.05 (0.74–1.49)	0.761	1.01 (0.71–1.44)	0.955	1.44 (0.95–2.18)	0.083	1.35 (0.88–2.05)	0.164
**≥ 1.0**	1.16 (0.80–1.67)	0.428	1.11 (0.77–1.60)	0.576	**1.83 (1.19–2.83)**	0.006	**1.71 (1.10–2.65)**	0.016
**High educational level**								
**< 0.1**	Ref.		Ref.		Ref.		Ref.	
**0.1–0.39**	1.21 (0.79–1.83)	0.373	1.15 (0.70–1.75)	0.528	1.36 (0.73–2.50)	0.330	1.27 (0.68–2.37)	0.446
**0.4–0.99**	**1.61 (1.07–2.42)**	0.022	**1.53 (1.00–2.32)**	0.046	**2.72 (1.48–5.00)**	0.001	**2.61 (1.38–4.94)**	0.003
**≥ 1.0**	**1.80 (1.19–2.73)**	0.005	**1.63 (1.08–2.48)**	0.0021	**1.98 (1.04–3.79)**	0.038	1.70 (0.86–3.36)	0.127

* Age-adjusted model of body mass index.

** Age adjusted model waist circumference.

OR = odds ratio; CI = confidence interval.

**Figure 2:**
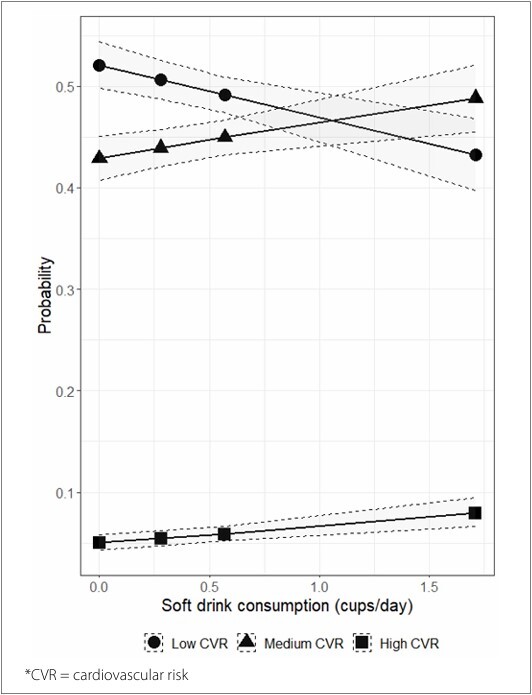
Conditional probability of low, medium, and high cardiovascular risk scores according to soft drink consumption levels, adjusted for body mass index and age. National Health Survey, 2013.

**Figure 2** presents the conditional probabilities of low, medium, and high CVR scores according to the level of consumption of soft drink servings. As consumption increased, the probability of a low CVR score decreased, while the probability of a high CVR score increased. 

## DISCUSSION

 Our data showed that consumption of sugar-sweetened soft drinks may increase the CVR scores in 10 years, with variations according to the quantity consumed, adjusted by age and anthropometric measures (BMI and WC), which are measures of adiposity highly associated with metabolic outcomes and chronic inflammation.^29^ The analyses also showed significance when stratified by race/color and educational level. The results presented here indicate that the daily consumption of one or more standard servings of soft drinks per day was independently and significantly associated with a greater probability of increased CVR scores. Similar results were observed in the subgroup analyses stratified by race/color and anthropometric measures. 

 The database used in this study was a representative subset of the Brazilian population, which mitigated the selection bias. The NHS was conducted between 2013 and 2014. Biological materials were collected from a subsample in 2014–2015 to perform laboratory tests on blood and urine samples. A survey in the Brazilian population with clinical data and biological fluid tests has never been conducted previously, which justifies its use despite being conducted in 2014. Thus, the estimate of CVR score in this study is considered representative of the Brazilian population.^17^ CVR estimates in other national studies were from restricted samples of clinical trials and specific populations.^33,34^ Measurement of CVR score has been used secularly in several previous and recent studies, which have demonstrated its validity.^20,21 ,35^

 In this study, soft drink consumption was measured through direct interviews. Participants were questioned about their usual consumption of sweetened soft drinks. Food consumption surveys are always susceptible to measurement bias, particularly memory bias. However, in most similar studies, the consumption of sweetened beverages was measured using questionnaires on the frequency of usual consumption, dietary history, or 24-hour recall. Beverage consumption levels were categorized by percentile distribution in standard serving units, using the no-consumption category as a reference. In this study, we adopted the methodology of a similar study on standard serving units.^16^

 Most studies in the literature are from cross-sectional, longitudinal, and meta-analysis studies, adding up to approximately 310,000 participants predominantly in White or Black populations in the United States, Finland, and China.^29^ Based on these studies, consumers who are in the highest quartile had a 20% and 26% excess risk of metabolic syndrome and diabetes, respectively, when compared to the lowest quartile of soft drink consumption.^29^ In a cross-sectional study not representative of the Brazilian population, the association between high consumption of soft drinks and the probability of metabolic syndrome was 95%.^16^ In a recent cohort study with a sample of 12,048 adults, consumption of 5–20 g/day of sugar (in this study a standard serving contains approximately 18.5 g of sugar) was associated with a risk of metabolic syndrome, particularly in women.^36^

 Two longitudinal studies have shown an increased risk of coronary heart disease with the consumption of soft drinks. In the cohort of 51,529 male health professionals aged 40–75 years, participants with consumption in the highest quartile compared with those in the lowest quartile had a 20% increased risk of coronary heart disease; these results were adjusted for appropriate confounding factors.^31^ Similarly, in another study, women from the *Nurse’s Health Study* showed a dose-response relationship between soft drink consumption and coronary heart disease; a median consumption of 1.2–2 standard servings/day, increased the risk of coronary heart disease by 23–25%, respectively.^32^ In the present study 27% and 21% of male and female participants, respectively, consumed ≥ 1 standard servings/day. Other studies have also suggested that high consumption of soft drinks is associated with weight gain, increased fat mass, hypertension, and a risky lipid profile,^11,29,37^ which in turn are also associated with increased CVR. 

 Typically, carbonated beverages in Brazil are sweetened with sucrose, a carbohydrate composed of glucose and fructose, causing high glycemic load that can potentiate the risk of metabolic diseases and increase the risk of metabolic deterioration.^9^ This metabolic impairment has been widely studied and associated with the development of cardiovascular involvement and type 2 diabetes.^38^

 The present study has some limitations. These designs considered the possibility of reverse causality, which could contradict the association between soft drink consumption and CVR. Additionally, insulin resistance indicators can be considered mediators of high sugar consumption and CVR. However, the magnitude of the association did not change when participants with glycated hemoglobin levels ≥ 6.5 or those receiving oral hypoglycemic agents or insulin were excluded. Another limitation is that national surveys are not designed to test specific hypotheses of association and are therefore susceptible to residual confounding. However, the greatest methodological advantage of this study lies in its ability to estimate the 10-year CVR scores representative of the Brazilian population— a population parameter rarely obtained by health surveys—which indicates the novel character of the results. 

 The results of this study suggest associative relationships, and not necessarily causal relationships, which are limited by the cross-sectional design of this investigation. However, the abundant literature in this area suggests a significant role of sugar consumption through beverages with added sugar, which may be a major source of calorie intake in the Brazilian population, with the aggravating factor of altering metabolic homeostasis. Thus, the findings of this study may help support public policies that aim to reduce the consumption of high-calorie processed foods. 

## CONCLUSION

 In this study, the association between soft drink consumption and 10-year CVR scores was shown to be independent of age and adiposity measures such as BMI and WC. Future studies using more robust methodologies, such as longitudinal studies and larger population samples, are required to better understand the underlying mechanisms, strengthen the scientific evidence, and inform effective public health policies. 

## References

[1] Previdelli ÁN, De Andrade SC, Pires MM (2011). A revised version of the healthy eating index for the Brazilian population. Rev Saude Publica.

[2] GBD 2019 Risk Factors Collaborators (2020). Global burden of 87 risk factors in 204 countries and territories, 1990-2019: a systematic analysis for the Global Burden of Disease Study 2019. Lancet.

[3] Elizabeth L, Machado P, Zinöcker M, Baker P, Lawrence M (2020). UltraProcessed Foods and Health Outcomes: A Narrative Review. Nutrients.

[4] Caprio S (2012). Calories from Soft Drinks — Do They Matter?. N Engl J Med.

[5] Brasil (2022). VIGITEL Brazil 2021 Surveillance of Risk and Protective Factors for Chronic Diseases by Telephone Survey.

[6] Canuto R, Fanton M, De Lira PIC (2019). Social inequities in food consumption in Brazil: A critical review of national surveys. Cien Saúde Colet.

[7] Amato D, Maravilla A, Garcia-Contreras F, Paniagua R (1997). Soft-drinks and health. Rev Invest Clin.

[8] Nomura K, Yamanouchi T (2012). The role of fructose-enriched diets in mechanisms of nonalcoholic fatty liver disease. J Nutr Biochem.

[9] Siqueira JH, Mill JG, Velasquez-Melendez G (2018). Sugar-sweetened soft drinks and fructose consumption are associated with hyperuricemia: cross-sectional analysis from the Brazilian Longitudinal Study of Adult Health (ELSA-Brasil). Nutrients.

[10] Johnson RJ, Segal MS, Sautin Y (2007). Potential role of sugar (fructose) in the epidemic of hypertension, obesity and the metabolic syndrome, diabetes, kidney disease, and cardiovascular disease. Am J Clin Nutr.

[11] Qin P, Li Q, Zhao Y (2020). Sugar and artificially sweetened beverages and risk of obesity, type 2 diabetes mellitus, hypertension, and allcause mortality: A dose-response meta-analysis of prospective cohort studies. Eur J Epidemiol.

[12] Siqueira JH, Pereira TSS, Moreira AD (2023). Consumption of sugarsweetened soft drinks and risk of metabolic syndrome and its components: results of the ELSA-Brasil study (2008-2010 and 20122014). J Endocrinol Invest.

[13] Denova-Gutiérrez E, Tucker KL, Flores M, Barquera S, Salmerón J (2016). Dietary patterns are associated with predicted cardiovascular disease risk in an urban Mexican adult population. J Nutr.

[14] Meng Y, Li S, Khan J (2021). Sugar- and artificially sweetened beverages consumption linked to type 2 diabetes, cardiovascular diseases, and all-cause mortality: a systematic review and dose-response metaanalysis of prospective cohort studies. Nutrients.

[15] Lobato JCP, Costa AJL, Sichieri R (2009). Food intake and prevalence of obesity in Brazil: an ecological analysis. Public Health Nutr.

[16] Velasquez-Melendez G, Molina MDCB, Benseñor IM (2017). Sweetened Soft Drinks Consumption Is Associated with Metabolic Syndrome: Cross-sectional Analysis from the Brazilian Longitudinal Study of Adult Health (ELSA-Brasil). J Am Coll Nutr.

[17] Szwarcwald CL, Malta DC, Souza PRB (2019). Laboratory exams of the National Health Survey: methodology of sampling, data collection and analysis. Rev Bras Epidemiol.

[18] Instituto Brasileiro de Geografia e Estatística (2014). Pesquisa nacional de saúde - 2013: percepção do estado de saúde, estilos de vida e doenças crônicas: Brasil, grandes regiões e unidades da federação.

[19] Szwarcwald CL, Malta DC, Pereira CA (2014). National Health Survey in Brazil: design and methodology of application. Cien Saúde Colet.

[20] D’Agostino RB, Vasan RS, Pencina MJ (2008). General cardiovascular risk profile for use in primary care: the Framingham Heart Study. Circulation.

[21] Malta DC, Pinheiro PC, Teixeira RA (2021). Cardiovascular risk estimates in ten years in the Brazilian population, a population-based study. Arq Bras Cardiol.

[22] Simão AF, Precoma DB, Andrade JP Á (2013). I Brazilian Guidelines for cardiovascular prevention. Arq Bras Cardiol.

[23] Instituto Brasileiro de Geografia e Estatística (2013). National Health Survey 2013-Tables.

[24] National Cholesterol Education Program (NCEP) Expert Panel on Detection, Evaluation, and Treatment of High Blood Cholesterol in Adults (Adult Treatment Panel III) (2002). Third Report of the National Cholesterol Education Program (NCEP) Expert Panel on Detection, Evaluation, and Treatment of High Blood Cholesterol in Adults (Adult Treatment Panel III) final report. Circulation.

[25] Barroso WKS, Rodrigues CIS, Bortolotto LA (2021). Brazilian Arterial Hypertension Guidelines – 2020. Arq Bras Cardiol.

[26] Williams B, Mancia G, Spiering W (2018). 2018 ESC/ESH Guidelines for the management of arterial hypertension. Eur Heart J.

[27] World Health Organization (2011). Use of Glycated Haemoglobin (HbA1c) in the Diagnosis of Diabetes Mellitus: Abbreviated Report of a WHO Consultation.

[28] American Diabetes Association (2018). 2. Classification and Diagnosis of Diabetes: Standards of Medical Care in Diabetes-2018. Diabetes Care.

[29] Malik VS, Popkin BM, Bray GA (2010). Sugar-sweetened beverages and risk of metabolic syndrome and type 2 diabetes: A meta-analysis. Diabetes Care.

[30] Mambrini SP, Menichetti F, Ravella S (2023). Ultra-processed food consumption and incidence of obesity and cardiometabolic risk factors in adults: a systematic review of prospective studies. Nutrients.

[31] De Koning L, Malik VS, Kellogg MD (2012). Sweetened beverage consumption, incident coronary heart disease, and biomarkers of risk in men. Circulation.

[32] Fung TT, Malik V, Rexrode KM (2009). Sweetened beverage consumption and risk of coronary heart disease in women. Am J Clin Nutr.

[33] Cesena FHY, Laurinavicius AG, Valente VA (2017). Cardiovascular risk stratification and statin eligibility based on the Brazilian vs. North American guidelines on blood cholesterol management. Arq Bras Cardiol.

[34] Bittencourt MS, Staniak HL, Pereira AC (2016). Implications of the New US Cholesterol Guidelines in the Brazilian Longitudinal Study of Adult Health (ELSA-Brasil). Clin Cardiol.

[35] Liu J, Hong Y, D’Agostino RB (2004). Predictive value for the Chinese population of the Framingham CHD risk assessment tool compared with the Chinese multi-provincial cohort study. JAMA.

[36] Pan F, Wang Z, Wang H (2022). Association between free sugars intake and risk of metabolic syndrome in Chinese adults: results from the China Health and Nutrition Survey, 2000-2018. Nutrients.

[37] Alcaraz A, Pichon-Riviere A, Palacios A (2021). Sugar sweetened beverages attributable disease burden and the potential impact of policy interventions: a systematic review of epidemiological and decision models. BMC Public Health.

[38] Dragsbæk K, Neergaard JS, Laursen JM (2016). Metabolic syndrome and subsequent risk of type 2 diabetes and cardiovascular disease in elderly women Challenging the current definition. Medicine.

